# Thermoplastic Elastomeric Composites Filled with Lignocellulose Bioadditives, Part 2: Flammability, Thermo-Oxidative Aging Resistance, Mechanical and Barrier Properties

**DOI:** 10.3390/ma13071608

**Published:** 2020-04-01

**Authors:** Justyna Miedzianowska, Marcin Masłowski, Krzysztof Strzelec

**Affiliations:** Institute of Polymer & Dye Technology, Lodz University of Technology, Stefanowskiego 12/16, 90-924 Lodz, Poland; marcin.maslowski@p.lodz.pl (M.M.); krzysztof.strzelec@p.lodz.pl (K.S.)

**Keywords:** thermoplastic elastomer blends, straw, waste management, mechanical and barrier properties, aging resistance, flammability

## Abstract

The work covers the characteristics of the functional properties of composites bordering thermoplastics and elastomers. The research is a continuation of considerations on blends in the form of a mixture of natural rubber (NR) with an ethylene–vinyl acetate copolymer (EVA) and the addition of a lignocellulose biofiller (wheat straw). After describing the processing and rheology as well as examining the thermal properties and morphology of composites (Part 1), the second part focuses on the characteristics of their performance. The effect of both different ratios of mixed polymers and the amount of filler on tensile strength and elongation at break, resistance to thermo-oxidative aging, hardness, tear resistance, barrier and damping properties, as well as flammability were investigated. The increased EVA content has shown a positive effect on tensile strength, elongation at break, resistance to thermo-oxidative aging, hardness, relative damping, tear strength, barrier and burning delay. On the other hand, a larger amount of natural rubber provides high flexibility and promotes the creation of a reinforcing structure by the filler used. Moreover, a significant impact of the addition of cereal straw on the barrier, damping, strength and flammability properties of composites was also noted. The great advantage of the prepared compositions in relation to commercial plastics is their environmental friendliness, primarily by replacing some petroleum derivatives of plastics with natural rubber and straw fibers.

## 1. Introduction

Thermoplastic elastomers (TPEs) constantly arouse the great interest of scientists, hence they are the subject of many studies and considerations described in the literature [1,2]. The goal of this research is always to obtain a material with the desired application properties, i.e., adapting an appropriate response to the growing needs and expectations of the industry. The field of TPEs involves all those products that must be able to return to their original shape after stress removal, but also to be able to be recycled [3]. TPEs are prepared by physical melt mixing of a polyolefin with an elastomer gained considerable attention because their macroscopic mechanical properties (impact strength, elongation at break) can be modified over a wide range by controlling the mixture composition [4,5,6]. Usually, blend ingredients are selected to complement both their pros and cons. For example, the main disadvantage of natural rubber is low aging resistance, and the EVA copolymer is characterized by high resistance. On the other hand, the flexibility of EVA itself may be too small for some applications, and the addition of NR significantly increases it.

The widespread use of polymers in the packaging, automotive, household appliances, electrotechnical and many other industries has led to several important problems. The biggest of them include growing pollution of the natural environment, depletion of raw materials for polymer synthesis and rising oil prices. Biocomposites, more environmentally friendly composites than traditional materials, can solve these problems [7,8,9]. In the case of biocomposites based on a synthetic polymer, fillers play the role of a natural component. An example is the starch [10], characterized by a low price, and the cellulose as a potential substitute for petrochemical polymers [11].

Fiber biofillers are becoming an increasingly frequent topic of research. The use of a filler in the fibrous form allows to additionally control the properties of composites in a wide range, depending on the selection and orientation of the fibers used [12,13]. Natural fibers have aroused great interest as a new type of thermoplastic elastomer filler because they increase strength and dissipate accumulated energy. At the same time, they are renewable, cheap, less destructive to processing systems and easy to modify chemically and physically [14,15]. In addition, natural fibrous materials have many advantages over glass or carbon fibers: low density, lower machine wear, high flexibility, non-toxicity, biodegradability, rigidity, availability from natural sources, renewable energy, efficiency and low cost [16,17,18]. Problems with the management of used plastics and the limited resources of substrates for their production meant that countless scientists are trying to replace them, at least in part, with materials of natural origin, creating, for example, biocomposites. Although natural fibers are one of the fastest growing polymer additives, the use of straw as a filler is still a relatively new and scientifically attractive idea [19,20,21,22].

The aim of the presented work was to obtain new biocomposites, materials of which properties are more favorable to nature and human activity than traditional materials. A composite consisting of a synthetic polymer and a natural filler is, therefore, an interesting solution in ecological and economic terms. The use of straw as a filler is also a potential solution to the problem of its management. The methods employed so far to use excess straw have some disadvantages and adding it to polymers and obtaining products with nondeteriorated and even better properties may be an interesting goal. When used in a traditional elastomer, however, the problem may be the high processing temperature required by the crosslinking process, which can lead to straw fiber degradation. The answer to this issue may be polymer mixtures which can be prepared at much lower temperatures than those for vulcanizates obtaining. The aspect was confirmed in the first part of this work on morphology, processing, thermal and rheological properties of such systems.

Based on previous studies, it has been confirmed that the application of the proposed technological and material solution causes significant benefits: no chemical crosslinking, reduction of processing temperature, prevention of fiber degradation, energy and economic savings. The present study, as a continuation of previous investigations, aimed to outline a set of new properties of the new biocomposites obtained and reported in the first part of this work. Thus, the biocomposites made of natural rubber (NR), ethylene-vinyl acetate (EVA) copolymer and lignocellulose biofiller (cereal straw) were found to exhibit improved performances by considering their mechanical characteristics, resistance to thermo-oxidative aging, flammability barrier and dumping properties.

## 2. Materials and Methods

### 2.1. Materials

• Polymer

Natural rubber (NR) RSS I was provided by Torimex-Chemicals (Lodz, Poland). Natural, polymeric product was obtained from rubber milk secreted by *Hevea brasiliensis* tress, with a density of 0.930–0.988 g/cm^3^ at 20 °C and a decomposition temperature below 200 °C (data based on safety data sheet).

Ethylene–vinyl acetate copolymer (EVA) known as EVA 1020 VN 5 with a vinyl acetate content of 17.5%, a melt flow index of 2 g/10 min (190 °C, 2.160 kg) and density of 0.94 g/cm^3^ was supplied by Total Petrochemicals (Houston, TX, USA).

• Fillers

Cereal (wheat, oat, rye, barley and triticale) straw was collected from Polish farms. Straw was dried and next crushed using a Blixer 4 Robot Coupe (Vincennes, France)—grinding time of 20 min. Then sieve analysis was performed by using: vibratory shaker, set of sieves with 2.0, 1.0, 0.5, 0.25 mm nominal mesh size. In the following studies, the fractions 0.5–0.25 and >0.25 mm were ground using a Fritsch Pulverisette 5 Classic Line planetary ball mill (Idar-Oberstein, Germany) for 2 h, a speed of 3000 rpm and a break of 30 min after 1 h. The compositions of NR/EVA and NR/EVA/straw are presented in Table 1. Each of the polymer mixtures was prepared in quantities such NR and EVA constituted 100 parts per hundred rubber.

### 2.2. Methods

Elastomer mixtures, based on natural rubber/ethylene–vinyl acetate copolymer and straw (Table 1), were prepared using an internal mixer (Brabender, Germany) at 80 °C with 50 rpm.

The polymer mixtures were formed using steel molds placed between the shelves of an electrically heated hydraulic press. The samples were pressed at 100 °C, at 15 MPa pressure for 10 min.

Mechanical properties of composites were measured by using a universal machine (Zwick, Ulm, Germany) in accordance with standard International Organization for Standardization (ISO) 37 by using standard dumbbell-shaped samples. The samples were subjected to a stress-strain test at a cross-head speed of 500 mm/min. The measurements were performed at room temperature. From the obtained stress-strain curve, tensile strength (TS) and elongation at break (Eb) were determined. The obtained results from the 5 specimens for each composite were averaged.

The thermo-oxidative aging characteristics were determined according to the PN-82/C-04216 standard. Samples were exposed to air at an elevated temperature (70 °C) for 14 days in a dryer FD (Binder, Tuttlingen, Germany) with thermo-circulation. To estimate the resistance of the blends to aging, their mechanical properties after aging were determined and compared with the values obtained for the blends before the aging process. The aging factor (K) was calculated as the numerical change in the mechanical properties of the samples upon aging (Equation (1)) [23]:

K = (TS · E_b_)_after aging_ /(TS · E_b_)_before aging_ (1)

The hardness of blends was determined according to ISO 868 standard using a Shore type A Durometer (Zwick, Ulm, Germany) and the results were averaged considering ten points randomly chosen for each sample.

Tear strength tests were carried out by a universal testing machine (Zwick, Ulm, Germany) in accordance with ISO 34 standard (dimensions of samples—100 mm × 15 mm, trouser pieces, test speed—50 mm/min). Measurements were repeated five times.

The relative damping of composites was measured according to the PN-C-04289 standard by using the disc-shaped samples, with the following dimensions: diameter—35 mm and height—17.5 mm. The analysis was carried out at room temperature by using a universal machine (Zwick, Ulm, Germany). Each sample was stressed from 0 to 0.7 MPa and then stress was gradually reduced. The loading–unloading cycle was repeated five times. Hysteresis loops were recorded and the relative damping values were determined according to the Equation (2) [24].

(2) $$T_{\tau w}=\frac{\Delta W_{i}}{W_{\mathrm{ibel}}}\cdot 100$$

where: T_τw_ is the relative damping (in %), ΔW_i_—the difference between the compression work and the work during reducing the compressive stresses and W_ibel_—compression work.

The flammability of blends was performed by, measuring the combustion time of samples (50 × 10 × 4 mm) in the air. The sample tip was ignited for 5 s by means of a gas burner supplied with propane–butane mixture.

Barrier properties were evaluated based on the through-plane air permeability of composites using the manometric method in accordance with the American Society for Testing and Materials (ASTM) D1434 standard. The tests were conducted by using atmospheric air at room temperature. Measurement of the barrier properties was based on a pressure difference between the chambers on both sides of the sample.

The barrier properties were expressed by gas transmission rate (GTR) and coefficient of gas permeability (P), according to the Equations (3) and (4) [25]:

(3) $$\mathrm{GTR}=\frac{V_{c}}{R\cdot T\cdot P_{u}\cdot A}\cdot \frac{\mathrm{dp}}{\mathrm{dt}}$$

(4) P=GTR·d

where d is the thickness of the sample [m], V_c_—volume of low-pressure chamber [L], T—temperature [K], P_u_—gas pressure in the high-pressure chamber [Pa], A—permeation area of gas through the sample [m^2^], dp/dt—pressure changes per unit time [Pa/s], R—gas constant 8.31·× 10^3^ (L·Pa)/(K·mol).

In the case of mechanical properties, i.e., tensile strength, tear strength, hardness, damping properties and flammability, a statistical analysis based on standard deviation was applied.

## 3. Results and Discussion

### 3.1. Mechanical Properties

**Figure 1:**
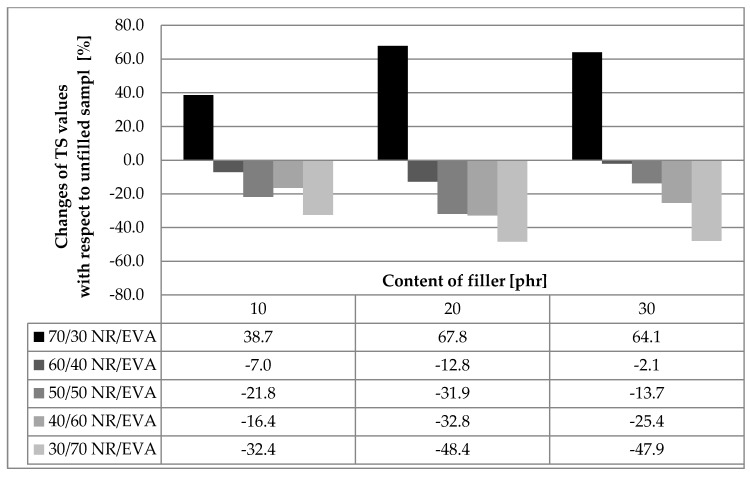
The percentage change in tensile strength for straw-based blend compared to unfilled systems.

The results of mechanical properties for the tested composites are presented in Table 2. In the case of unfilled blends, the tensile strength increased with increasing EVA content in the composite. The reason for the low mechanical strength at high relative elongation is the low degree of physical crosslinking due to the EVA phase in glassy-crystalline state at room temperature. Thus, the TS value of the NR/EVA 70/30 composite was only 3.5 MPa, and the composite with inverse polymer proportions was over three times higher—12.0 MPa. On the other hand, from the strengthening point of view, the addition of straw in the case of samples with the same or preponderant content of EVA copolymer with respect to rubber content was proved to be unfavorable. Embedding 10 phr of straw into the NR/EVA 30/70 composite reduced the TS value by 32%. The addition of straw component to 20 and 30 phr reduced TS by as much as about 50% compared to the unfilled system (Figure 1). However, for samples NR/EVA 40/60 and NR/EVA 50/50, the negative effect of the biofiller was already much smaller, ranging from 16 to 32% (Table 1 and Figure 1). Composites containing more NR had initially less strength, but the addition of straw led to the most beneficial when compared to the corresponding straw-free blends. Sample NR/EVA 70/30, which had the lowest TS value without filler, after the addition of 20 and 30 phr straw was already as strong as samples containing more EVA with the same degree of filling. The large differences in the strength of unfilled composites substantially decreased as a result of the addition of almost the same values around 6 MPa (see Table 1 and Figure 1).

The same type of straw of similar shape, size and susceptibility to agglomeration was used in all filled blends. One of the parameters affecting the mechanical strength of composites is the filler—polymer interactions. The tensile strength results indirectly prove that at higher straw content, their dispersion in the polymeric matrix is optimal and the biofiller makes eventually a reinforcing structure into the studied ternary blends. Accordingly, the filler–polymer interactions are favorable enough to avoid straw aggregation/agglomeration into larger particles and, at the same time, the strength of the filler–filler interactions were enough to create percolation pathways that may carry any potential applied stress.

The highest elongation at break (ca. 800–900%) was achieved by composites containing a larger amount of NR (Table 2). A characteristic feature of natural rubber obtained even after crosslinking is its very high Eb values. The use of straw reduced the flexibility and, consequently, Eb values for all the prepared biocomposites as its volume/weight content raised. This effect was observed for almost all filled systems. Under the influence of external stresses, elastomer chains may easily undergo huge deformations as a result of large degree of freedom available for the macromolecular segments. The presence of biofiller particles diminishes the free volume associated to elastomeric component, reducing its ability to lightly change its dimensions on molecular scale.

### 3.2. The Thermo-Oxidative Aging Process

The resistance of the prepared biocomposites to thermo-oxidative aging has been determined based on changes in their mechanical properties. Analyzing the results of tensile strength and relative elongation of blends before and after aging (Table 2), it was noted that at the highest EVA content of the tricomponent systems, the TS values decreased by 20–25% almost regardless of the amount of straw. The tensile strength of the samples with the highest NR content was reduced by half. As expected, increasing the percentage of EVA in the blends enhanced the resistance to thermo-oxidative aging of the entire systems. The beneficial effect of increased EVA content in composites to the aging resistance of systems was also visible when comparing Eb values. The elongation at break of NR/EVA 30/70 samples was reduced by 1–18% and by 20–40% for the systems with the reverse proportion.

**Figure 2:**
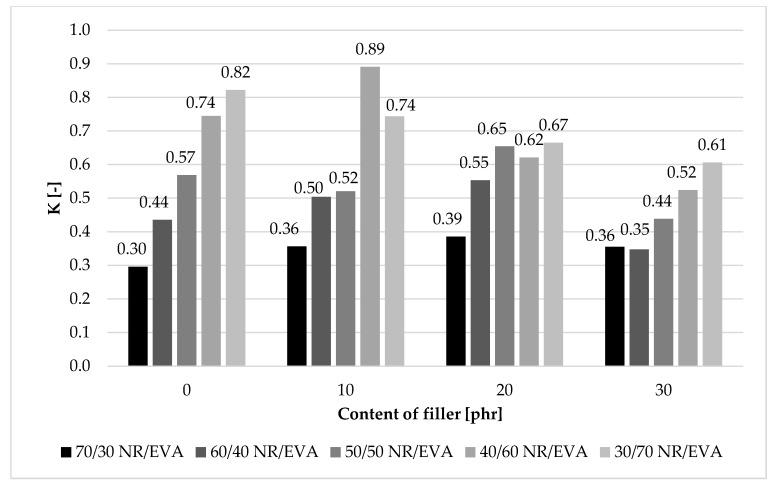
The thermo-oxidative aging factor of blend natural rubber/ethylene–vinyl acetate copolymer blends.

In order to more easily determine changes in the deformation energy of composites after simulated thermo-oxidative aging processes, the aging coefficient K was calculated. It is a numerical measure of the composite’s resistance to aging. The closer its value to 1, the smaller the changes in the tested properties before and after aging. The lowest aging rate was observed for composites containing 70 and 60 phr of EVA copolymer, filled with 10 phr of straw (Figure 2). For example, the beneficial effect (10 phr) of straw was particularly evident in the 40/60 NR/EVA composite, where the K value increased by 0.20 compared to the K value for NR/EVA 30/70 composites. In the case of blends with a natural rubber/EVA copolymer ratio of 70/30 and 60/40, the addition of straw to 10 and 20 phr had a positive effect on the composite’s resistance to thermo-oxidative aging. The increase of the K coefficient caused by the addition of straw could be due to the presence of lignin. Its structure includes polyphenols, which are well-known as natural antioxidants. More specifically, they are primary antioxidants that provide an electron or hydrogen atom to the reactive polymer radicals [26]. So, the final products can be either non-radical or radical species, the latter being stable radicals, which may interrupt degradation of polymeric components via free-radical mechanism during aging process. Another reason for slowing down the aging process could be the increased barrier of the material. The rate of deterioration of polymer properties as a result of exposure to temperature, light and other external conditions is closely dependent on the rate of oxygen diffusion into the material. With a proper fiber dispersion, oxygen penetration through the interface between the NR/EVA structure and crystalline cellulose regions is hindered, leading to the formation of a fewer free radicals. Nevertheless, it should be considered that, apart from polyphenols, the biofiller contains a lot of aging-sensitive compounds, such as cellulose. Too much filler in the composite led to the deterioration of the composites’ aging resistance. The composites with the addition of straw showed lower thermal stability (Part 1) because an amount of the NR/EVA components was replaced by straw with a relatively low decomposition temperature. In this case, the overall thermal energy needed to induce chemical transformations was much smaller, so the initiation of thermo-oxidation occurred easier and faster.

### 3.3. Damping Properties

**Figure 3:**
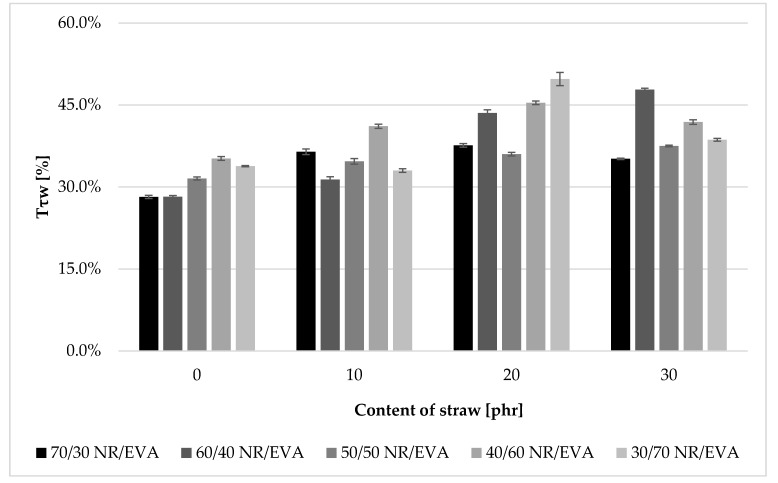
The relative damping factor of unfilled and filled with straw natural rubber/ ethylene–vinyl acetate copolymer (NR/EVA) blends.

The relative damping of filler-free composites was around 30–35% (Figure 3). For straw-filled composites, the Tτw value falls within the range of 35–50%. One of the highest relative damping values was obtained for composites NR/EVA 40/60 and 30/70 with the addition of 20 phr straw. Equally, high damping was demonstrated by sample NR/EVA/straw 60/40/30. Considering the results for all blends, the addition of straw increased the relative damping value, most for 20 phr content. In composites without the addition of straw, the higher content of the EVA copolymer had a beneficial effect on the value of damping coefficient. NR/EVA 40/60 composites were characterized by the highest vibration absorption capacity. For the filler-based samples, the effect of EVA did not obey a monotone tendency. Thus, higher values of dumping coefficient were observed for both the ratio of NR to EVA 40/60, 30/70 and 60/40.

According to the elastic solid theory, under the influence of external forces, material particles move from the original state of equilibrium to another. This movement is counteracted by internal forces and if they are large, the displacement is small. After removing the external forces, internal forces tend to restore, without heat loss, the particles to their original position and the deformation is is pure. The main feature of elastic materials is the large deformability (shortening angle, deflection) in the direction of the force. In this way they accumulate potential energy, absorb vibrations and mitigate impacts. In addition, elastic elements into a viscous matrix not only receive but also dissipate energy. Rubber parts with sufficiently high damping ability can work in various drives and even support steel structures and systems. Compounds filled with straw, especially with 20 phr and/or containing more EVA, would work best as vibration-absorbing and impact-absorbing materials. Such materials would be able to accumulate a lot of potential energy without any damages on them.

### 3.4. Hardness

**Figure 4:**
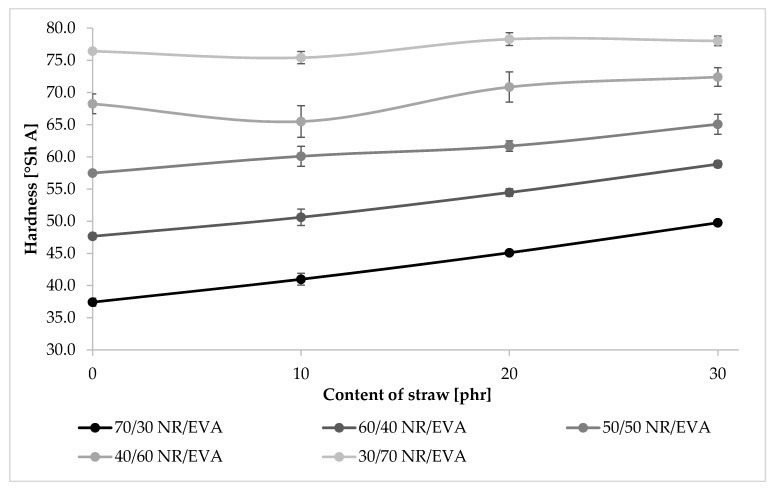
The hardness values of biocomposites.

The hardness of all samples was in the range of 37–78 Shore A degrees (Figure 4). With the increase in EVA content, its value also increased, almost in a linear way. For samples containing 70 phr of NR and 30 phr of EVA, the hardness was about 40. In the case of samples with the inverse proportion, the hardness increased almost twice as a result of the higher content of the crystalline phase. The addition of straw also increased the hardness by several units for each of the tested blends. The reason for the higher hardness, in this case, was also the increase in the content of the crystalline phase because the cellulose that builds the straw fibers has a high degree of order.

### 3.5. Tear Strength

**Figure 5:**
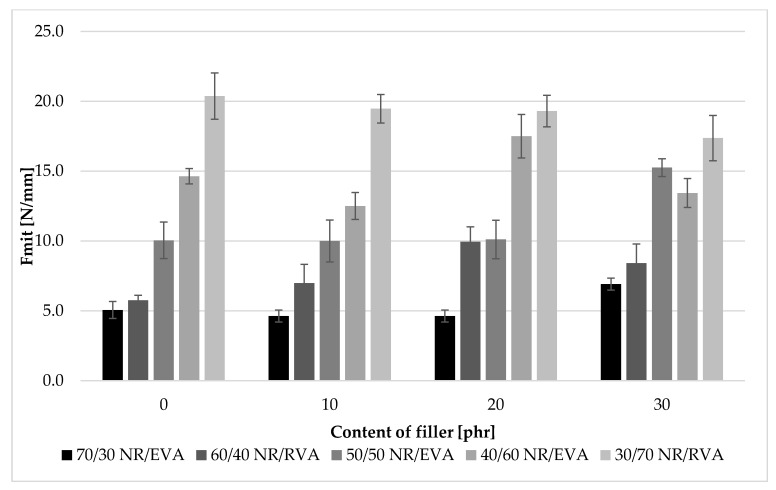
The average strength needed to tear the sample (Fmit) of NR/EVA or NR/EVA/straw composites.

The study showed that there was a close relationship between EVA content and blend tear resistance (Figure 5). The higher the copolymer content, the greater the force needed to break the sample. The F_mit_ value of NR/EVA 30/70 samples was about 4 times higher than the NR/EVA 70/30 blend, almost regardless of the presence of straw. Samples containing more rubber had a tear strength of 5–7 N/mm, and with increasing EVA content, the strength increased up to 20 N/mm. The intermolecular interactions associated to the copolymer component (cohesive forces), as a result of its crystalline/glassy state at room temperature, are substantially greater than those within natural rubber microdomains and represents an appreciable energy barrier for incision propagation. The addition of straw also increased the tear resistance of composites. On average, the highest values were obtained for the addition of 20 phr of straw. In such a proportion, the fibers create a tear-limiting barrier that, when crossed, requires more force to be applied.

### 3.6. Flammability

Measurement of the burning time of samples in the air allowed to assess the effect of the amount of polymers (NR, EVA) and straw on the flammability of composites (Table 3). As expected, EVA slowed the burning of composites, but the effect was visible only for the ternary blends with 20 and 30 phr of straw. The combustion time of NR/EVA 30/70 samples filled with 20 and 30 parts by weight of straw was longer by over 1.5 min than of NR/EVA 70/30 samples with the same degree of filling. A relatively surprising result was the extension of the burning time of the samples due to straw filling. Although the straw itself burned quickly and with a large flame, its addition to the polymer composite significantly slowed down the burning of the entire system. For example, embedding an amount of 10 phr of straw extended the burning time of the NR/EVA 70/30 sample from 219 to 351 s (60%) and for NR/EVA 40/60 from 215 to 301 s (40%). Increasing the amount of straw extended the burning time by several dozen seconds for samples NR/EVA 40/60 and 30/70. Sample NR/EVA/straw 30/70/30 burned the longest, almost 7.5 min, while the shortest—unfilled composite NR/EVA 50/50, 3.5 min.

**Figure 6:**
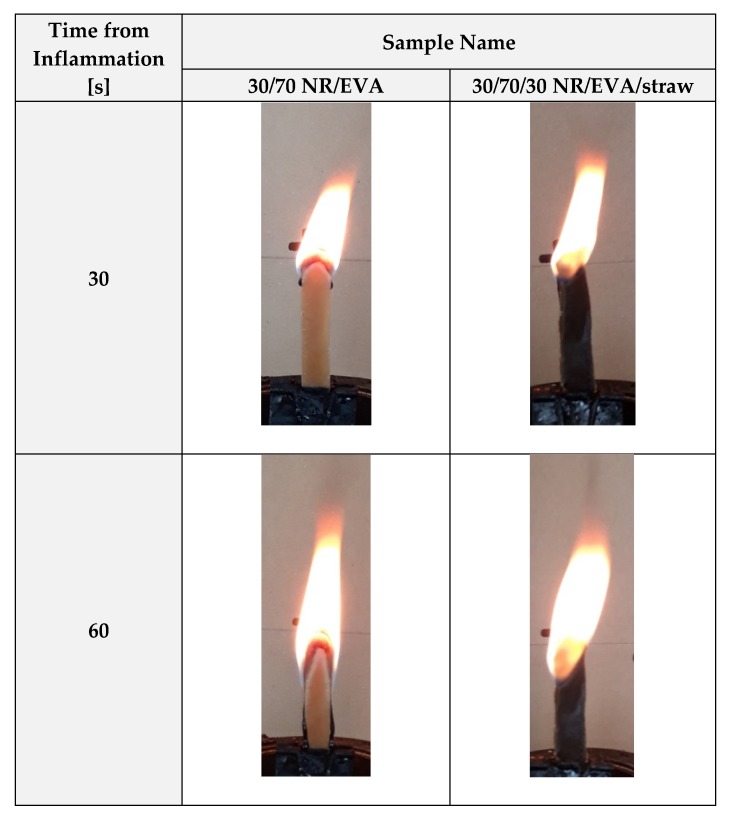
The comparison of sample behavior during combustion for 30/70 NR/EVA (unfilled and filled with 30 phr straw).

During the tests, differences in the manner of burning individual materials were also observed. First of all, the unfilled blend samples dripped, and the flame spread with the flowing drops. In addition, the flow of melted polymer droplets was higher at higher ethylene-vinyl acetate copolymer contents in the blend. This is due to the fact that EVA has a relatively low melting point and lower viscosity than NR, which makes it quicker to flow. In the event of a fire, the dripping phenomenon is extremely dangerous because the flowing drops transfer the flame to nearby elements and the area of the fire increases. More favorable results in terms of flame retardancy were obtained for straw-filled composites, especially for 20 and 30 phr of filling. These samples did not drip, and the flame that covered them at the same stages of smoking was clearly smaller. The method of smoking and the difference in flame size between samples without and with straw addition observed 30 and 60 s after the ignition are shown in Figure 6.

The reason for slowing down the composite burning was probably a decrease in air permeability and thus limited oxygen diffusion into the burning sample. The increase in barrier properties of composites with the addition of straw has been proven in air permeability studies. In addition, the spread of liquid polymer combustion products has been eliminated. The higher the straw content in the composite, the lower the gas permeation coefficient and the lower the permeability were observed. Straw fibers formed areas in the polymer matrix that are difficult to avoid by gas molecules. For this reason, oxygen diffusion into the polymer blends was slower. Smoking strictly depends on the amount of oxygen available, therefore limiting its amount has slowed down the combustion process. In addition, straw increased the viscosity of the material, which significantly reduced the spreading of the liquid material and affected the burning time. It is also very possible that the liquid EVA copolymer, instead of flowing down the sample, has been absorbed by straw fibers.

The study showed that among composites filled with 10 phr lignocellulosic material, samples with a higher content of NR were less flammable. In contrast, blends containing 20 and 30 phr of biofiller burned longer if they contained more EVA than NR. In terms of burning retardation, the most advantageous was the use of 30/70, 40/60 and 50/50 NR/EVA composites filled with 20 and 30 parts by weight of a straw. Potentially, they could be used as LSZH (Low Smoke, Zero Halogen) materials due to the lack of halogens and the significant slowdown in smoking compared to pure NR. Typically, such materials are filled with inorganic additives, limiting the amount of diffusing oxygen, increasing thermal capacity and affecting the rheology of the polymer. These types of fillers undergo endothermic decomposition, absorb heat from the polymer and produce inert gases that reduce the concentration of free radicals conditioning the maintenance of the combustion reaction [27]. Although straw is an organic material, some of these effects most likely occur when used in composites.

### 3.7. Barrier Properties

The barrier properties of the obtained blends were significantly different depending on the ratio of the polymers used to form the polymer matrix as well as the amount of bio-filler used (Table 4). To exemplify, the gas transmission rate (GTR) for the material made of NR/EVA/biofiller 70/30/10 reached the highest value of 2.04·10^−8^ mol/(m^2^∙s∙Pa). The study carried out for composites with a lower natural rubber content revealed a huge impact of EVA fraction on the gas transmission rate of blends. Even a small change in the ratio NR/EVA from 70/30 to 60/40 led to reducing the gas transmission rate by up to 80%. Further increasing the amount of EVA in the blend resulted in a decrease in sample permeability, but the differences were less significant.

The addition the straw also exhibited a positive effect on the barrier properties of composites. The changes caused by its addition were not as large as when increasing the EVA content, but nonetheless observable. In almost all cases, the transmission rate gradually decreased as the straw content increased. The greatest effect of the filler on the rate of air permeation through the composite was observed with the addition of 30 phr for composite NR/EVA 40/60. The GTR value for this blend decreased from 3.25·× 10^−9^ to 2.09·× 10^−9^ mol/(m^2^∙s∙Pa), which is a difference of 34% compared to the reference system. The improved barrier properties of the blend resulted from the greater crystallinity of the EVA copolymer (in conjunction with the glassy state of copolymer at room temperature), hence from the greater orderliness and stability of the structure limiting the gas penetration of the material. The surprising result is the significant difference between the samples NR/EVA 70/30 and NR/EVA 60/40 and relatively small between samples containing more and more copolymer. This effect is most likely related to the percolation theory: the probability that the gas will find its way from one side of the sample to the other depends on the number of paths available to its molecules.

Analyzing the results of air permeability through composites (P) considering the thickness of the sample, it could be seen that the addition of straw reduced the permeability, to the greatest extent for the sample NR/EVA 40/60. The value of P as a result of adding 30 parts by weight decreased almost by half from 4.64 × 10^−12^ to 2.49 × 10^−12^ mol·m/(m^2^·s·Pa). The filler in this amount also had a large impact on the permeability of samples with a 60/40 and 50/50 rubber to copolymer ratio—a reduction of about 30%. In the NR/EVA 70/30 sample, the reduction was smaller—13%, and in the NR/EVA 30/70 composite—the smallest (ca. 4–12%). In the filled composites, gas molecules must travel a longer distance in the polymer matrix. The dispersed straw fibers create further barriers that the gas must bypass, which limits its diffusion. This effect can be compared with a maze, the narrower the paths and turns, the more difficult it is to get to the other side. The more straw fibers, the more barriers and the more complicated the maze. This was in line with the results obtained, which showed that a larger amount of straw resulted in the greater barrier. High barrier, so the smallest possible gas permeation coefficient for a given material, is crucial for most applications of polymeric materials. This is one of the main properties that determine such wide use of plastics, e.g., in the packaging industry and construction. Therefore, reducing the rate of gas permeation through the addition of straw and copolymer is very desirable.

The paper presents research on thermoplastic elastomers in the form of blends made of ethylene–vinyl acetate and natural rubber with different mass fractions of these polymers. In addition, natural fibers (ground cereal straw) were introduced into these systems, which allowed to increase the share of raw materials of natural origin in the composite, bring material benefits, and propose the possibility of their utilization. When introducing new technological solutions, it is extremely important to recognize and characterize newly created materials.

To sum up the whole work (Part 1 and 2), it should be emphasized that newly formed composites are combining the advantages of natural rubber and EVA copolymer and partly eliminating their disadvantages (e.g., high aging susceptibility of NR). Produced NR/EVA blends with a co-continuous structure were characterized by high flexibility while maintaining strength at the appropriate level. The homogeneous dispersion of the straw filler has contributed to a positive effect on a number of functional properties such as barrier, flammability, damping and mechanical strength. The ecological aspect related to the use of natural, renewable, agricultural waste was also significant. In addition, due to the appropriate processing conditions, cross-linking of composites has been eliminated, which facilitates material recycling and its re-use. From the point of view of material engineering, the innovative NR/EVA/straw blends present high application potential, create the possibility of using straw surpluses and fit into the trend of sustainable development principles.

## 4. Conclusions

The proposed blends are characterized by interesting and diverse properties depending on the material composition. Blends containing a predominance of ethylene-vinyl acetate copolymer were characterized by reduced elongation at break, while increased tensile strength. It is noteworthy that straw has reduced tensile strength in these systems. In turn, in the case of the larger mass friction of natural rubber in blends, the straw was able to create a more extensive secondary structure, with a significant strengthening character. The biocomposites produced were characterized by varied resistance to thermo-oxidative aging. The increased EVA content in the blend allowed to obtain composites with higher resistance to degradation under the influence of increased temperature. However, the impact of straw depended on its quantity and type of blend. The hardness at the highest EVA content almost doubled, and the addition of straw also increased it. Tests on damping properties have shown that composites containing more EVA and filler can find potential applications in finished products that absorb vibrations and mitigate impacts. The positive effect of the combination of natural rubber with the ethylene-vinyl acetate copolymer as well as the straw was also observed on the basis of tear strength results. The addition of ethylene-vinyl acetate copolymer to natural rubber also had a huge impact on the barrier properties of the material. Furthermore, straw fibers have also increased the barrier properties of composites. The fibrous nature of the filler used has resulted in a structure that significantly extends the gas diffusion path through the sample. The flammability test of composites also brought promising results. Among samples filled with 10 phr straw, those with a higher NR content were less flammable. The straw contained in the composite contributed to the absorption of liquid decomposition products and prevented the spread of flame, which is an extremely advantageous aspect from the point of view of polymer materials exposed to fire.

## Figures and Tables

**Table 1:** The composition of polymer mixtures.

Sample Name	Natural Rubber	Ethylene-Vinyl Acetate	Straw
	[phr]		
**70/30**	70	30	0
**70/30/10**			10
**70/30/20**			20
**70/30/30**			30
**60/40**	60	40	0
**60/40/10**			10
**60/40/20**			20
**60/40/30**			30
**50/50**	50	50	0
**50/50/10**			10
**50/50/20**			20
**50/50/30**			30
**40/60**	40	60	0
**40/60/10**			10
**40/60/20**			20
**40/60/30**			30
**30/70**	30	70	0
**30/70/10**			10
**30/70/20**			20
**30/70/30**			30

Phr—Parts Per Hundred Rubber.

**Table 2:** The mechanical properties of blends before and after the aging process.

Sample	Content of Filler [phr]	Before Aging		After Aging	
		TS [MPa]	Eb [%]	TS [MPa]	Eb [%]
**NR/EVA 70/30**	0	3.5	843	1.7	510
	10	4.9	825	2.5	573
	20	5.9	712	3.1	524
	30	5.8	614	2.6	490
**NR/EVA 60/40**	0	6.3	833	3.6	630
	10	5.8	704	3.5	583
	20	5.5	552	3.7	452
	30	6.1	525	3.0	368
**NR/EVA 50/50**	0	7.4	795	5.2	647
	10	5.8	638	4.1	473
	20	5.0	481	3.8	412
	30	6.4	438	4.1	300
**NR/EVA 40/60**	0	8.5	762	7.1	678
	10	7.1	621	6.2	636
	20	5.7	463	4.6	352
	30	6.3	426	4.6	307
**NR/EVA 30/70**	0	12.0	756	10.0	748
	10	8.1	589	6.5	549
	20	6.2	460	4.6	416
	30	6.3	432	4.6	354

**Table 3:** The flammability of composites.

Total Burning Time [s]				
NR/EVA	Content of Filler [phr]			
	0	10	20	30
**70/30**	219	351	317	327
**60/40**	227	346	327	303
**50/50**	209	335	350	344
**40/60**	215	301	350	382
**30/70**	254	310	411	445

**Table 4:** The barrier properties of polymer blends based on natural rubber and ethylene–vinyl acetate copolymer filled with straw.

**P ∙ 10^11^ [mol/(m ∙ s ∙ Pa)]**				
**NR/EVA**	**Content of Filler [phr]**			
	**0**	**10**	**20**	**30**
**70/30**	2.34	2.86	2.32	2.04
**60/40**	0.64	0.50	0.47	0.44
**50/50**	0.53	0.36	0.37	0.39
**40/60**	0.46	0.41	0.31	0.25
**30/70**	0.25	0.24	0.22	0.24
**GTR ∙ 10^8^ [mol/(m^2^ ∙ s ∙ Pa)]**				
**NR/EVA**	**Content of Filler [phr]**			
	0	**10**	**20**	**30**
**70/30**	1.91	2.04	1.68	1.60
**60/40**	0.44	0.42	0.36	0.35
**50/50**	0.41	0.31	0.30	0.28
**40/60**	0.32	0.31	0.23	0.21
**30/70**	0.21	0.20	0.18	0.18
